# The bidirectional relationship between head injuries and conduct problems: longitudinal modelling of a population-based birth cohort study

**DOI:** 10.1007/s00787-023-02175-y

**Published:** 2023-02-24

**Authors:** Hannah R. Carr, James E. Hall, Hedwig Eisenbarth, Valerie C. Brandt

**Affiliations:** 1 School of Psychology, Centre for Innovation in Mental Health, University of Southampton, University Road, Highfield Campus, Building 44, Southampton, SO17 1PS UK; 2 Southampton Education School, University of Southampton, Southampton, SO17 1BJ UK; 3 School of Psychology, Victoria University of Wellington, Wellington, 6140 New Zealand

**Keywords:** Conduct problems, Head injury, Cross-lagged path model, Cumulative risk index, Developmental psychopathology

## Abstract

Childhood head injuries and conduct problems increase the risk of aggression and criminality and are well-known correlates. However, the direction and timing of their association and the role of their demographic risk factors remain unclear. This study investigates the bidirectional links between both from 3 to 17 years while revealing common and unique demographic risks. A total of 8,603 participants (50.2% female; 83% White ethnicity) from the Millennium Cohort Study were analysed at 6 timepoints from age 3 to 17. Conduct problems were parent-reported for ages 3 to 17 using the Strengths and Difficulties Questionnaire (SDQ) and head injuries at ages 3 to 14. A cross-lagged path model estimated the longitudinal bidirectional effects between the two whilst salient demographic risks were modelled cumulatively at three ecological levels (child, mother, and household). Conduct problems at age 5 promoted head injuries between 5 and 7 (*Z* = 0.07; *SE* = 0.03; 95% CI, 0.02–0.13), and head injuries at ages 7 to 11 promoted conduct problems at age 14 (*ß* = .0.06; *SE* = .0.03; 95% CI, 0.01–0.12). Head injuries were associated with direct child-level risk at age 3, whereas conduct problems were associated with direct risks from all ecological levels until 17 years. The findings suggest a sensitive period at 5–11 years for the bidirectional relationship shared between head injuries and conduct problems. They suggest that demographic risks for increased head injuries play an earlier role than they do for conduct problems. Both findings have implications for intervention timing.

**Supplementary Information:**

The online version contains supplementary material available at 10.1007/s00787-023-02175-y.

## Introduction

Childhood conduct problems and head injuries are both significant risk factors for lifelong aggression and criminality [1, 2] and are known correlates [3]. However, how and when conduct problems and head injuries increase the other during childhood, particularly when controlling for demographic risk factors, remains unknown. This poses a serious problem for professionals in health, social care, and education. Without knowing when and to what extent head injuries pose a risk for conduct problems (and vice versa) it is difficult to design and deploy interventions with the greatest potential for impact.

Conduct problems can be defined as repeated violations to age-appropriate societal norms [4], such as fighting, threatening, and bullying. One of the potential causes of conduct problems is head injury [5]. Head injury is the main cause of death and disability in the UK, with approximately 1.4 million admissions of head injury every year, of which 33–55% are children [6].

Clinical studies have shown increased conduct problems following traumatic brain injuries (TBI) [5, 7]. Mild head injuries (those that do not disrupt normal brain functioning) are similarly associated with increased odds of delinquent behaviours at ages 11 and 14 [8], and with greater levels of conduct problems in adolescence and early adulthood [9]. Mechanisms explaining how head injuries pose a risk for increased conduct problems include changes to brain areas linked with executive functioning. In particular, over-activation of attention networks [10] and changes in neural connectivity resulting in task switching difficulty [11].

Research, however, investigating conduct problems influence on the risk of head injuries is limited. Studies typically investigate this relationship alongside ADHD [12] or from adolescent to adulthood [13]. However, a recent study suggests that childhood conduct problems at age 5 can similarly predict an increased risk of sustaining head injuries from ages 7 to 11 [3]. Mechanisms to explain this association similarly include changes to brain areas. For example, the ventral striatum (associated with reward processing) has been shown to be impaired in those with conduct problems and is related to greater real-life risk-taking [14]. Such risk-taking may provide a greater opportunity to sustain a head injury including through rough and tumble play, which has been shown to be more common in those with conduct problems [15].

Although the current literature suggests a potential bidirectional association between childhood conduct problems and head injury, no published study explicitly investigated this association, nor identified a sensitive age in which these associations take place. This information is critical to inform effective interventions. Limitations of many previous studies is their focus on TBIs, while 95% of head injuries are mild or never reported [6], the inclusion of clinical samples, self-reported head injuries, long delays in reporting of head injuries, and failure to control for common factors influencing both conduct problems and head injuries. We sought to account for such limitations by investigating whether there is a bidirectional association between head injuries and conduct problems during child development from 3 to 17 years in a large, longitudinal UK cohort. Importantly, the current study controls for salient demographic risk factors concerning the child, their mother, and their household, leading to two research questions:

1. Are there bidirectional associations between head injuries and conduct problems from ages 3 to 17 years?
2. Is combined risk at the child, mother and household levels associated with conduct problems and/or head injuries from ages 3 to 17 years?

## Methods

### Study design and participants

Participants were part of the Millennium Cohort study (MCS), a longitudinal birth cohort study of 18,786 individuals born in the UK between 2000 and 2002 [16]. They were studied at seven time points, at 9 months (T1), 3 (T2), 5 (T3), 7 (T4), 11 (T5), 14 (T6), and 17 years (T7). Analyses were limited to those with complete conduct problem data at the last wave (T7) [17, 18, 19]. Further exclusions were made to those who were not first-born children to allow independence of observation[20] and due to different levels of aggression related schemas and head injury risk in siblings [21, 22]. Final exclusions were made to those whose main respondent was not their biological mother as the focus of mother-related risk (see below) such as mother to child attachment were measured only for the biological mother. This resulted in an analytic sample of 8,603 individuals (4,322 female [50.2%]; 83% White ethnicity; see flow chart in Supplementary Fig. 1).

All procedures and analyses were approved by the University of Southampton Ethics Committee (ID = 62,100). Families provided written informed consent to take part and consented for their data to be shared for secondary analysis. Data were downloaded from the UK Data Archive [beta.ukdataservice.ac.uk/datacatalogue/series/series?id = 2000031].

### Measures

#### Conduct problems

These were assessed from age 3 (T2) using the five items from the Conduct Problem Subscale of the parent-report version of the Strengths and Difficulties Questionnaire (SDQ) [23]. Items are scored on a 3-point scale (0—2) with a higher total score indicating a higher level of conduct problems (possible range: 0—10). Cronbach’s alpha values within this study ranged from 0.52 to 0.66 across the MCS waves. Previous research has shown the SDQ to have over 75% sensitivity in identifying clinically relevant conduct problems [24], the parent version specifically has strong validity in identifying conduct disorder [25], and has been shown to be invariant across timepoints [26].

#### Head injuries

Parents were asked if their child had ever, or since the last wave, sustained a head injury that resulted in them being taken to the doctor, health centre, or hospital. Head injuries (coded 1) included responses categorised as a ‘bang on the head’ or ‘loss of consciousness’. The ‘loss of consciousness’ group was extremely small meaning that there would not have been the statistical power to warrant analysing the groups separately. The overall ‘head injury’ variables also capture everyday head injuries sustained in the general population as opposed to the moderate-severe head injuries that are often the focus of the literature. Head injury data was analysed from T2 onwards to achieve temporal ordering with the studies risk factors.

#### Demographic risks

Demographic risks were divided by ecological level (child, mother, and household) and combined risk from each level was measured via a cumulative risk index (CRI). Each CRI consisted of five items dichotomised into 0’s (low risk) and 1’s (high risk) based on the literature and summated. A higher score indicated the presence of more risks in a child’s development. Further details of each CRI can be seen in Supplementary Table 1.

#### Child level risk

Child level risk factors were taken from the parent interview at T1 and included male sex [2, 27], low birth weight (< 2.5 kg) and premature birth (< = 252 days gestation) [28, 29], and whether the child’s biological mother smoked or drank alcohol during pregnancy [30].

#### Mother level risk

Mother level risk factors were from the parent interview at T1 and included pregnancy before 18 years [27, 31], no high-school qualification [30, 31, 32], current unemployment [30], low attachment with child (< = 22 on Condon Maternal Attachment Scale) [33, 34], and psychological distress (> 4 on Rutter Malaise Inventory) [27, 35].

Attachment with child was measured using a subset of six items from the Condon Maternal Attachment Questionnaire [33]. The items were scored on a scale from 1 (*almost all the time*) to 5 (*never*; possible range: 0—30). A lower score indicates greater difficulties in mother–child attachment. Maternal psychological distress was measured using the MCS’s 9-item composite variable of the Rutter Malaise Inventory’s original 24-item scale [35]. The items were coded as 0 (*no)* and 1 (*yes)* and summed (possible range: 0 – 9) with a higher score indicating higher psychological distress.

#### Household level risk

Household level risk factors were taken from the parent interview at T1 and T2. These included single parent household [31, 36], low household income (< 60% of median household income) [31, 36], household overcrowding (fewer rooms than people excluding bathrooms and hallways) [31, 36], low household occupational status (highest occupational status in the household being semi-skilled or lower) [32], and a low-quality home learning environment (bottom quartile of early home learning environment scale) [37]. The home learning environment was measured at T2 using six items used in the home learning environment scale available in the MCS dataset (excluding ‘*playing with numbers’*) [37]. These measured the frequency at which the child engaged in learning activities. These items were scored on a rating-scale from 0 (*not at all*) to 7 (*everyday*) and summed (possible range 0—42). A higher score indicates a higher quality home learning environment.

#### Covariates

#### ADHD

ADHD was controlled for due to its high comorbidity with conduct problems [38] and its high association with sustaining a head injury [12]. ADHD was measured from age 5 (T3) to age 14 (T6) by asking the parent if their child had a diagnosis of ADHD. A binary variable was generated (0 = no diagnosis, 1 = diagnosis of ADHD).

#### Epilepsy

Epilepsy was controlled for due to its increased association with sustaining a head injury [39]. Epilepsy was measures from age 3 (T2) to age 17 (T7) by asking the parent if their child had a diagnosis of epilepsy. A binary variable was generated (0 = no diagnosis, 1 = diagnosis of epilepsy).

### Statistical analysis

**Fig. 1:**
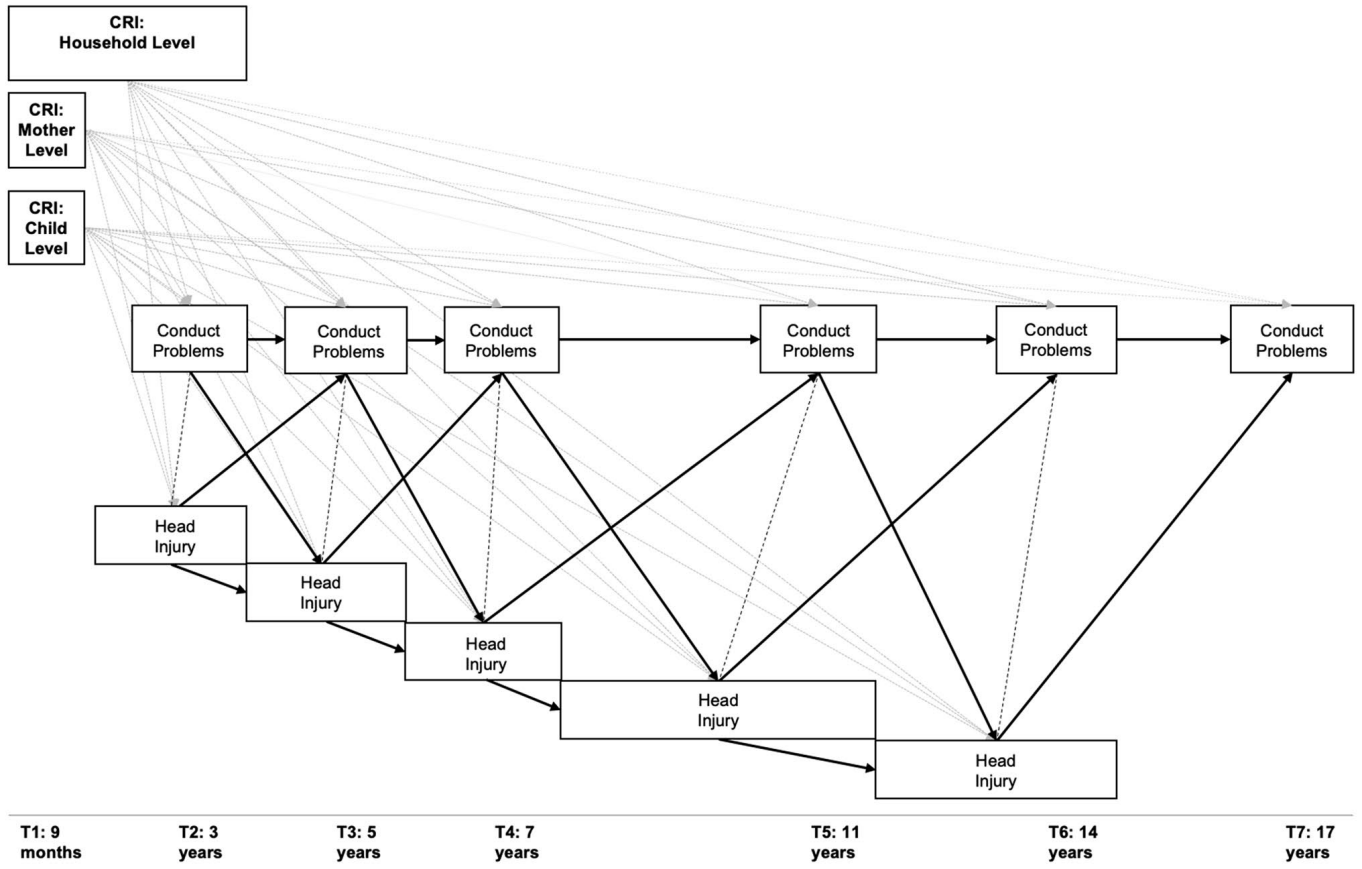
Stylized Illustration of the Structural Equation Model Implemented in This Study. This figure shows the cross-lagged path model conducted on conduct problem variables from age 3 (T2) to 17 (T7) and head injury variables from age 3 (T2) to 14 (T6). These are connected by contemporaneous correlations as well as lagged paths to T + 1 within and across variables. The three cumulative risk indices (CRI) at the child, mother, and household-levels are connected to each head injury and conduct problem variable (dotted lines). Solid lines represent pathways between conduct problem and head injury variables. Dashed lines represent correlations within timepoints

Mplus (v7.4) was used to run a cross-lagged path model (see Fig. 1) to test the relationships between head injury and conduct problems over time while controlling for salient demographic risks, ADHD, and epilepsy. MCS sample weights from T7 were applied to account for stratification, attrition, and nonresponse bias. The internal validity of the statistical estimates concerning the binary head injury variables were improved through use of the weighted least square estimation procedure. Missing data were accounted for through the use of the Full Information Maximum Likelihood procedure.

Contemporaneous correlations were included to account for the relationship within-timepoints [40]. As the correlations were between a binary and continuous variable, Mplus calculated point-biserial (*r*_*pbis*_) correlations.

Total, direct, and indirect effects were modelled and reported (see Primer for further information on these effects [41]). Indirect effects (e.g., the indirect effect of T2 head injuries on T4 conduct problems via T3 head injuries) were reported as total indirect effects (sum of all indirect effects). However, where a total indirect was not significant but an individual indirect effect was, the individual indirect effect was reported.

Model fit was evaluated based on the following criteria: Tucker-Lewis Index (TLI; acceptable fit ≥ 0.90, good fit ≥ 0.95), the Comparative Fit Index (CFI; acceptable fit ≥ 0.90, good fit ≥ 0.95), and Root Mean Square Error of Approximation (RMSEA; acceptable fit < 0.08, good fit < 0.05) [42, 43]. Where conduct problems (continuous) were the dependent variable, standardised beta values (*ß*) were reported. Where head injury (binary) was the dependent variable, the standardised *Z*-value (index of probit regression) was reported. Results were considered significant with *α* = 0.05.

### Data availability

The MCS dataset used in this study is available via the UK Data Service. The Mplus output for the direct and indirect effects as well as the code needed to create the CRI variables can be accessed via Pure.

## Results

### Participants and demographics

Table 1 provides a summary and comparison of sample characteristics between the excluded and analytical samples. The samples differed significantly on all variables, though these effects were weak (Cramér’s V < 0.20, Cohen’s *d* < 0.20). A breakdown of the head injury variable can be seen in Supplementary Table 2.

**Table 1:** Characteristics of and Differences Between the Analytical (*n* = 8,603) and Excluded Sample (*n* = 10,183)

	Analytical (*n* = 8,603)		Excluded (*n* = 10,183)				
Variable	*N* (%)	Mean (SD)	*N* (%)	Mean (SD)	Chi-square (df)	*p*	Cramér’s V
Sex					14.80 (1)	< 0.01	0.3
Male	4,281 (49.8)	–	5,354 (52.6)	–	–	–	–
Female	4,322 (50.2)	–	4,829 (47.4)	–	–	–	–
Ethnicity					15.69 (5)	0.08	0.3
White	7,137 (83)	–	8,354(82)	–	–	–	–
Mixed	246 (2.9)	–	316 (3.1)	–	–	–	–
Black	265 (3.1)	–	413 (4.1)	–	–	–	–
Indian	222 (2.6)	–	248 (2.4)	–	–	–	–
Pakistani	602 (7)	–	669 (6.6)	–	–	–	–
Other	117 (1.4)	–	149 (1.5)	–	–	–	–
Conduct problems							
Age 3	7,648 (88.9)	2.69 (2.00)	6,710 (65.9)	2.95 (2.12)	7.59 (14,356)^a^	< 0.001	0.13^b^
Age 5	7,965 (92.6)	1.42 (1.46)	6,428 (63.1)	1.61 (1.56)	7.40 (14,391)^a^	< 0.001	0.12^b^
Age 7	7,812 (90.8)	1.29 (1.48)	5,338 (52.4)	1.52 (1.63)	8.34 (13,148)^a^	< 0.001	0.15^b^
Age 11	7,971 (92.7)	1.28 (1.49)	4,430 (43.5)	1.56 (1.68)	9.50 (12,399)^a^	< 0.001	0.18^b^
Age 14	7,798 (90.6)	1.33 (1.57)	3,259 (32)	1.57 (1.72)	7.14 (11,055)^a^	< 0.001	0.15^b^
Age 17	8,603 (100)	1.17 (1.48)	770 (7.6)	1.24 (1.55)	1.21 (9,371)^a^	0.225	0.05^b^
Head injuries							
9 months-3 years	1,012 (11.8)	–	857 (8.6)	–	50.12 (1)	< 0.001	0.05
3–5 years	761 (8.8)	–	624 (6.8)	–	43.93 (1)	< 0.001	0.05
5–7 years	573 (6.7)	–	384 (3.8)	–	73.64 (1)	< 0.001	0.06
7–11 years	496 (5.8)	–	271 (3.1)	–	107.33 (1)	< 0.001	0.08
11–14 years	386 (4.5)	–	120 (1.5)	–	186.77 (1)	< 0.001	0.10

If (n) is less than the n included, this refers to missing data within the variable

^a^Independent samples *t* test

^b^Cohen’s *d*

### Association between head injury and conduct problems across development

The cross-lagged path model showed acceptable fit (χ^2^(32) = 468.34; *p* < 0.001; RMSEA = 0.02 [0.018, 0.022]; CFI = 0.93; TLI = 0.84) with all except the TLI meeting the predefined acceptable threshold [42, 43].

The contemporaneous correlations between head injury and conduct problems were small, positive, (*r*_*pbis*_ < 0.10) and significant (*p* < 0.05) at age 3 (T2) and 17 (T7).

**Fig. 2:**
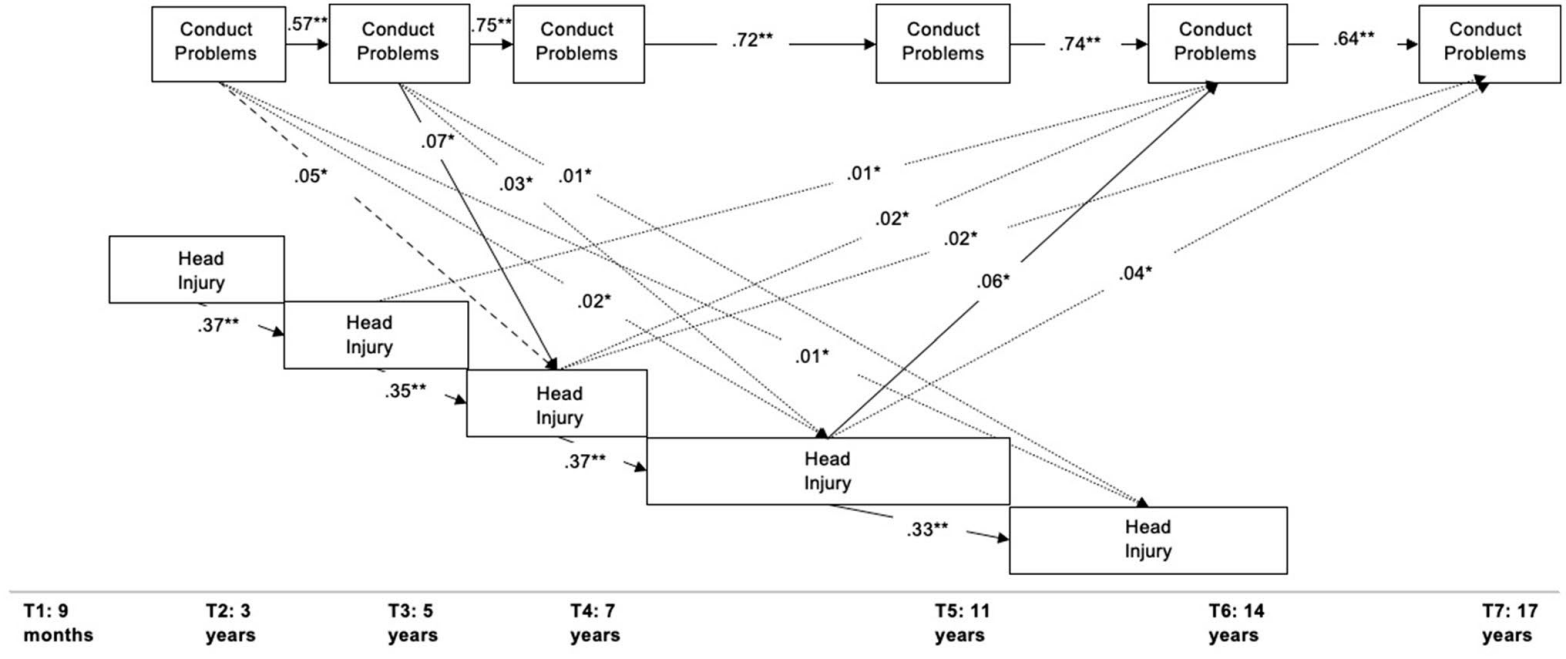
The Direct and Indirect Effects Within and Between Conduct Problems and Head Injury From Ages 3 to 17. This figure shows the significant direct effects (solid lines) within and between the head injury and conduct problem variables, the significant total indirect (dashed lines), and the individual indirect (dotted lines) effects. All indirect effects from head injury to later head injury variables (T + 1 onwards) and from conduct problems to later conduct problem variables were significant but omitted for clarity. Only significant pathways are shown to *p* < 0.05 (*) and *p* < 0.001 (**)

Head injury at each time point had significant direct (Fig. 2, Supplementary Table 3) and indirect effects (Fig. 2, Supplementary Table 4) for an increased likelihood of subsequent head injury, as did conduct problems for increased subsequent conduct problems (Fig. 2, Supplementary Tables 3 and 4).

Head injury at ages 7 to 11 had a direct effect for increased conduct problems at age 14 (*ß* = 0.06; *SE* = 0.03; 95% CI 0.01–0.12). Head injuries at ages 3 to 5 and 5 to 7 had significant individual indirect effects linked to greater conduct problems at age 14 (*ß* = 0.01; *SE* = 0.004; 95% CI 0.001–0.02; *ß* = 0.02; *SE* = 0.01; 95% CI 0.002–0.05 respectively). Head injuries at ages 5 to 7 and 7 to 11 had significant individual indirect effects linked to greater conduct problems at age 17 (*ß* = 0.02; *SE* = 0.01; 95% CI 0.002–0.03; *ß* = 0.04; *SE* = 0.02; 95% CI 0.01–0.08, respectively). See Fig. 2 for visualisation.

Conduct problems at age 5 had a direct effect for an increased likelihood of head injury between ages 5 and 7 (*Z* = 0.07; *SE* = 0.03; 95% CI 0.02–0.13). There were significant total indirect effects from conduct problems at age 3 for an increased likelihood of head injuries at ages 5 to 7 (*Z* = 0.05; *SE* = 0.02; 95% CI 0.01–0.08). Significant individual indirect effects were identified from conduct problems at ages 3 and 5 for an increased likelihood of head injuries at ages 7 to 11 (*Z* = 0.02; *SE* = 0.01; 95% CI 0.003–0.03; *Z* = 0.03; *SE* = 0.01; 95% CI 0.01–0.05, respectively) and of head injuries at ages 11 to 14 (*Z* = 0.01; *SE* = 0.002; 95% CI 0.001–0.01; Z = 0.01; *SE* = .0.004; 95% CI 0.001–0.02, respectively)). See Fig. 2 for visualisation.

### The influence of child, mother and household-level demographic risk factors

Child-level cumulative risk had a significant direct effect for increased conduct problems at ages 3, 5, 11, and 17 (Table 2, Supplementary Fig. 2). Mother-level cumulative risk had a significant direct effect for increased conduct problems at ages 3, 5, 7, and 17 (Table 2, Supplementary Fig. 3). Household-level cumulative risk had a significant direct effect for increased conduct problems at ages 3, 11, and 17 (Table 2, Supplementary Fig. 4). All three CRIs had significant total indirect effects for increased conduct problems from ages 5 to 14 (Table 2, Supplementary Figs. 2, 3, and 4) and significant individual indirect effects for age 17 (Child: *ß* = 0.07, *SE* = 0.02, 95% CI 0.04 –0.10; Mother: *ß* = 0.02, *SE* = 0.004, 95% CI 0.02–0.03; Household: *ß* = 0.05, *SE* = 0.01, 95% CI 0.03–0.08).

**Table 2:** The total, direct, and total indirect effects of the child, mother and household CRIs on conduct problems and head injury

	Total effect^a^	SE	95% CI	Direct effect^a^	SE	95% CI	Total Indirect effect^a^	SE	95% CI
Conduct problems									
Age 3									
Child CRI	–	–	–	0.14**	0.02	0.11–0.17	–	–	–
Mother CRI	–	–	–	0.12**	0.02	0.109–0.16	–	–	–
Household CRI	–	–	–	0.25**	0.02	0.22–0.28	–	–	–
Age 5									
Child CRI	0.15**	0.02	0.10–0.19	0.07*	0.02	0.02–0.11	0.08**	0.01	0.06–0.10
Mother CRI	0.16**	0.02	0.13–0.20	0.09**	0.02	0.06–0.13	0.07**	0.01	0.05–0.09
Household CRI	0.18**	0.02	0.14–0.22	0.04	0.02	< 0.001–0.07	0.14**	0.01	0.12–0.16
Age 7									
Child CRI	0.06*	0.03	0.01–0.11	− 0.05	0.03	− 0.11–0.01	0.11**	0.02	0.08–0.14
Mother CRI	0.20**	0.02	0.15–0.23	0.07**	0.02	0.03–0.10	0.12**	0.01	0.10–0.15
Household CRI	0.15**	0.03	0.10–0.20	0.02	0.03	− 0.03–0.07	0.13**	0.01	0.11–0.16
Age 11									
Child CRI	0.19**	0.03	0.13–0.23	0.14**	0.03	0.08–0.20	0.04*	0.02	0.01–0.08
Mother CRI	0.09**	0.02	0.05–0.13	− 0.04	0.02	− 0.09–0.001	0.14**	0.01	0.11–0.16
Household CRI	0.22**	0.02	0.17–0.26	0.11**	0.03	0.06–0.16	0.11**	0.02	0.07–0.15
Age 14									
Child CRI	0.08	0.05	− 0.03–0.18	− 0.07	0.06	− 0.19–0.05	0.15**	0.02	0.10–0.19
Mother CRI	0.19	0.20	− 0.19–0.58	0.12	0.21	− 0.27–00.51	0.07**	0.02	0.04–0.10
Household CRI	0.03	0.10	− 0.17–0.24	− 0.13	0.12	− 0.34–00.08	0.17**	0.02	0.13–0.20
Age 17									
Child CRI	0.13*	0.04	0.05–0.21	0.08*	0.03	0.03–0.14	0.05	0.04	− 0.02–0.12
Mother CRI	0.16	0.12	− 0.07–0.40	0.04*	0.02	0.002–0.08	0.12	0.13	− 0.12–0.37
Household CRI	0.07	0.06	− 0.05–0.18	0.05*	0.02	0.004–0.09	0.02	0.07	− 0.11–0.15
Head injuries									
9 months–3 years									
Child CRI	–	–	–	0.08*	0.03	0.02–0.14	–	–	–
Mother CRI	–	–	–	− 0.001	0.03	− 0.06–0.06	–	–	–
Household CRI	–	–	–	− 0.03	0.03	− 0.09–0.03	–	–	–
Age 3–5									
Child CRI	0.05	0.03	− 0.01–0.11	0.02	0.03	− 0.03–0.08	0.03*	0.01	0.01–0.05
Mother CRI	0.01	0.03	− 0.05–0.07	0.01	0.03	− 0.05–0.06	0.002	0.01	− 0.02–0.02
Household CRI	− 0.03	0.03	− 0.09–0.04	− 0.02	0.03	− 0.08–0.05	− 0.01	0.01	− 0.03–0.02
Age 5–7									
Child CRI	0.05	0.03	− 0.003–0.11	0.03	0.03	− 0.03–0.08	0.03*	0.01	0.01–0.05
Mother CRI	− 0.04	0.03	− 0.10–0.03	− 0.05	0.03	− 0.11–0.01	0.02	0.01	− 0.01–0.04
Household CRI	− 0.03	0.03	− 0.09–0.03	− 0.03	0.03	− 0.10–0.03	0.004	0.01	− 0.02–0.03
Age 7–11									
Child CRI	0.17*	0.08	0.03–0.32	0.15	0.08	− 0.001–0.31	0.02	0.01	− 0.004–0.04
Mother CRI	0.05	0.06	− 0.07–0.17	0.07	0.07	− 0.06–0.20	− 0.02	0.01	− 0.04–0.01
Household CRI	0.09	0.06	− 0.03–0.20	0.10	0.06	− 0.02–0.22	− 0.01	0.01	− 0.04–0.01
Age 11–14									
Child CRI	0.09*	0.04	0.02–0.16	0.03	0.04	− 0.06–0.12	0.06*	0.03	0.01–0.11
Mother CRI	− 0.05	0.05	− 0.15–0.04	− 0.07	0.05	− 0.17–0.03	0.02	0.02	− 0.02–0.06
Household CRI	− 0.02	0.06	− 0.13–0.10	− 0.05	0.06	− 0.16–0.07	0.03	0.02	− 0.01–0.07

*SE* standard error*; CRI* cumulative risk index; *CP* conduct problems; *HI* head injury

^a^If dependent variable is CP then standardized beta coefficient (ß) is reported if HI then the standardized z-value coefficient is reported

*p < 0.05

**p < 0.001

Only the child-level cumulative risk had a significant direct effect for an increased likelihood of head injuries from 9 months to 3 years (see Table 2, Supplementary Fig. 2). Total indirect effects were significant only at the child-level for head injuries sustained at ages 3 to 5, 5 to 7, and 11 to 14 (Table 2, Supplementary Fig. 2). However, significant individual indirect effects were present from the mother and household-levels to head injuries sustained at ages 5 to 7 (*Z* = 0.01, *SE* = 0.002; 95% CI = 0.001–0.01; *Z* = 0.01, *SE* = 0.004; 95% CI = 0.002–0.02, respectively)), and the household-level for ages 7 to 11 (*Z* = 0.004, *SE* = 0.002; 95% CI = 0.001–0.01).

## Discussion

The aim of this study was to identify if there were bidirectional associations between conduct problems and head injuries in a UK population between the ages of 3 and 17 years, while controlling for salient demographic risk factors. The results showed that higher levels of conduct problems at age 5 promoted an increased likelihood of head injury between the ages of 5 to 7 whilst a head injury sustained between the ages of 7 and 11 promoted increased conduct problems at age 14. Thus, this study shows a longitudinal, bidirectional relationship between head injuries and conduct problems during a sensitive period between the ages of 5 and 11 years. Further, the bidirectional relationship between head injury and conduct problems exists over and above the effects of salient demographic risk factors at the child, mother, and household-level as well as ADHD and epilepsy.

These results provide further evidence that childhood head injuries are associated with increased levels of conduct problems [2]. However, it elaborates on the previous literature by suggesting that this relationship is bidirectional and that conduct problems also promote head injuries during the sensitive period of 5 to 11 years. This was only previously identified when there was a co-morbid diagnosis of ADHD [12] or in a young adult population [13]. This clarifies results shown by Brandt and colleagues [3] whilst controlling for salient demographic risk. Thus, the current study provides novel insight into a potential bidirectional association between head injury and conduct problems that warrants further investigation.

In line with existing literature, child, mother, and household demographic risks all had direct and indirect effects for increased conduct problems over the course of development (from 3 to 17 years) [27, 30, 31]. However and surprisingly, the mother and household risks were found to play no direct role in promoting head injuries during childhood (from age 9 months). Direct risk instead lied solely at the level of the child with all but one of these risk factors (male sex) being themselves socially stratified.

### Strengths and limitations

The key strength of the current study is its use of a large birth cohort dataset which enabled the statistical unpacking of the complex relationships linking conduct problems to head injuries and vice versa over time. Another strength is the comprehensive inclusion of all head injuries, which increases the ecological validity to the findings.

A limitation of this paper is the stringent inclusion criteria for participants, which limited the generalizability of the findings to the general UK population. The analytical sample differed from the total sample on demographics including ethnicity. Therefore, the results may not reflect the ethnic diversity within the UK population meaning that these results must be read with caution. The analytical sample also had significantly lower mean conduct problems than the total sample, suggesting that the sample may not be representative of conduct problems presented in the general UK population.

Parent-report for both head injuries and conduct problems might be considered a limitation. Though this addresses the limitations of previous head injury research whereby self-report is likely to inhibit accuracy (i.e. due to infantile amnesia) [44], it could introduce a social desirability bias. Therefore, this research (as with all research using parent measures) requires smaller-scale follow-up using more objective measures, such as clinical records.

### Implications

Parents and teachers may work together to identify those children with high levels of conduct problems when they enter primary school as these children are at an increased risk for sustaining a head injury. This is particularly important as this is a critical developmental period where children enter school and begin to have reduced parental supervision and increased peer interaction. All of which could result in greater opportunities to sustain a head injury. Additional safety precautions may be administered in schools to try to counteract the increased risk for head injuries, which poses a subsequent risk for an increase in conduct problems until age 14.

Examples include limiting or prohibiting contact sports where there is the potential to sustain a head injury [45] and interventions to encourage helmet usage when riding a bike [46].

## Conclusions

The results of this study suggest a sensitive period between the ages of 5 and 11 where conduct problems and head injuries are risk factors for one another with consequences for interventions that run both before and during this period.

### Supplementary Information

Below is the link to the electronic supplementary material.

Supplementary file1 (DOCX 372 KB)
